# May-Thurner Syndrome: A Suspicion That Led to an Incidental Discovery

**DOI:** 10.7759/cureus.33862

**Published:** 2023-01-17

**Authors:** Ahmed Kazi, Abeera Akram, Brooj Awan, Carolina Borz-Baba

**Affiliations:** 1 Internal Medicine, Saint Francis Hospital, Hartford, USA; 2 Internal Medicine, Saint Mary's Hospital, Waterbury, USA

**Keywords:** pulmonary emboli, iliac compression syndrome, endovascular thrombectomy, deep vein thrombosis (dvt), endovascular stent graft

## Abstract

May-Thurner syndrome (MTS)/Iliac vein compression syndrome is characterized by left iliac vein stenosis secondary to compression by the right common iliac artery against the fifth-lumbar vertebra. It increases the incidence of deep venous thrombosis (DVT). We had a 43-year-old female presenting with left leg swelling and was found to have extensive DVT provoked by a long history of travel. Because of the extension of her thrombus, a catheter-guided thrombectomy (CDT) was planned and findings of MTS were identified incidentally. She had an endovascular stent placed and was discharged on long-term anticoagulation.

## Introduction

May-Thurner syndrome (MTS) is one of the anatomical variant syndromes that impedes the venous flow in the iliac vein by extrinsic arterial compression, frequently in the presence of a vertebral process. The incidence of MTS varies significantly from 2%-5% to 25% [1] and it is considered commonly overlooked as a cause of left lower extremity deep venous thrombosis (DVT), particularly in young patients. We describe a case of a patient with extensive ipsilateral thrombosis due to MTS, and we discuss the risk factors and the diagnostic and therapeutic challenges associated with this unusual condition.

## Case presentation

A 43-year-old lady arrived at the emergency department reporting 24 hrs of severe left leg pain and swelling that started after fourteen hours of non-stop driving. The pain was preceded by a sensation of leg heaviness that the patient attributed to prolonged sitting; it made ambulation extremely difficult. The patient did not report skin lesions or tingling of the left lower extremity, shortness of breath, coughing up blood, or chest pain. The patient had no past medical or surgical history, including no history of miscarriages. She had never used oral contraceptives. The patient denied smoking and did not use illicit drugs. She had no family history of malignancy, clotting disorders, or miscarriages. Her initial vitals were a pulse rate of 119 beats/minute, blood pressure of 141/89 mm Hg, saturating 100% on room air with a respiratory rate of 16, and temperature of 98.3 F. Physical examination was remarkable for significant edema with warmness and extreme tenderness of the left leg extending from the groin to the shin. Examination of the right lower extremity was normal. The pulses were symmetrically palpable in both legs.

**Figure 1:**
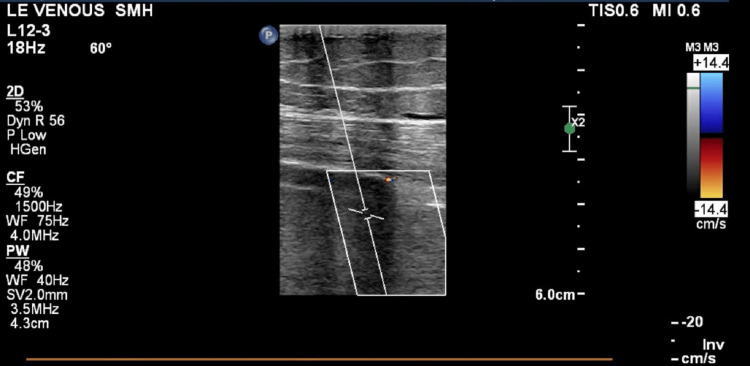
Left leg Duplex venous ultrasound exhibiting no venous flow

Initial laboratory results revealed hemoglobin of 11.0 g/dl, sodium 128 mEq/L, chloride 93 mEq/L, creatinine of 0.8 mg/dl, PTT 31 sec, PT 13.5 sec and INR 1.1. Duplex venous ultrasound of the lower extremity identified an extensive occlusive thrombus extending from the left common iliac veins (LCIV) to the proximal calf veins (Figure 1). No venous flow was noted in the LCIV and the patient was started on a heparin drip. Computed tomography angiography (CTA) of the chest revealed small right lower lobe and left lower lobe segmental pulmonary emboli. The extent of the thrombus increased the probability of a massive iliofemoral DVT. The case was discussed with the hematology and the interventional radiology service (IR). To investigate a potential MTS and to prevent significant residual thrombus and likely a post-thrombotic syndrome, the patient was referred for catheter-directed thrombectomy (CDT) and venography.

**Figure 2:**
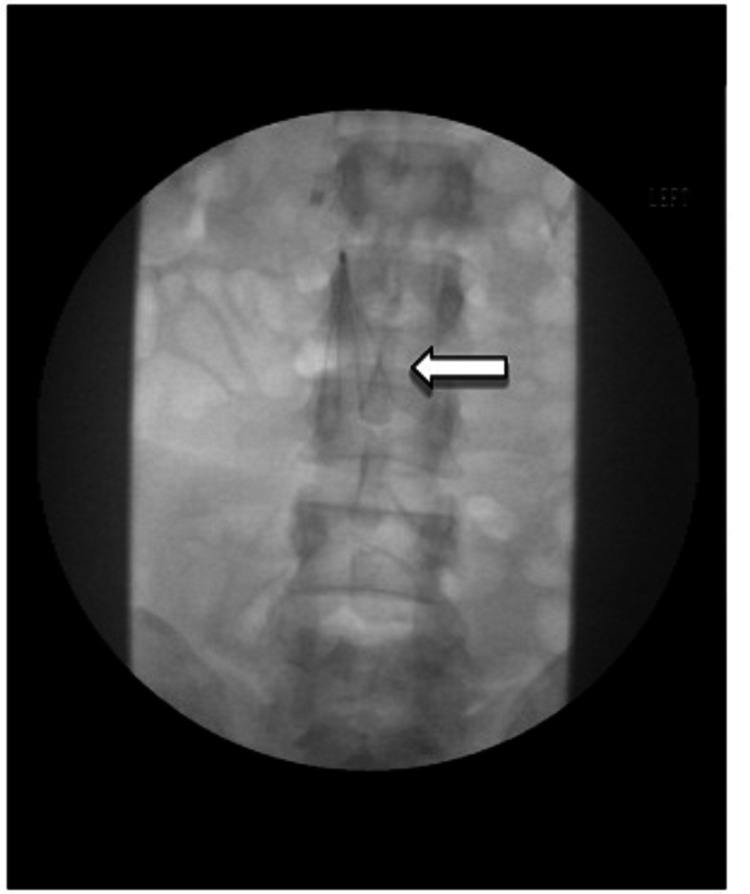
Inferior vena cava filter in place

The procedure revealed that the thrombus extended from the left common iliac origin into the lateral aspect of the mid to distal inferior vena cava (IVC) lumen. No definite flow was identified from the LCIV. A prophylactic IVC filter was placed for the extent of the procedure (Figure 2). A multi-hole intravenous catheter was advanced from the LCIV to the distal IVC. Catheter guided thrombolysis was planned and tissue plasminogen activator infusion was initiated locally at the rate of 0.7 mg/hour. The peripheral heparin infusion rate was increased to 300 units/hour. Post-procedure, the patient was transferred to the intensive care unit for overnight monitoring. The next day, a significant residual occlusive thrombus was observed in the LCIV, and a subtotal clearing of the thrombus in the left femoral vein was identified. Successful thrombectomy of residual thrombi was achieved. The subsequent venography demonstrated a high-grade stricture at the proximal LCIV consistent with MTS.

Successful stenting of the LCIV restored brisk anterograde flow and the procedure concluded with the removal of the IVC filter which was placed as a bridge to avoid any thromboembolic event.

The patient’s symptoms resolved after the recanalization of the left iliac vein and she was able to ambulate without pain. She was discharged home on long-term oral anticoagulation (rivaroxaban) and lifelong aspirin with a follow-up with her primary care physician and hematologist.

## Discussion

MTS was claimed by doctors May and Turner in 1957 following their investigations of the thrombosis of the pelvic veins. The pathophysiology of iliac vein compression syndrome remains intricated. The most postulated hypothesis is related to developing a spur in the venous wall secondary to the chronic pulsation of the overlapping right iliac artery followed by collagen depositions. Chronic compression may contribute to local intimal fibrosis, a nidus for thrombosis. An underlying inherited hypercoagulable state is commonly demonstrated in patients with MTS, adding to the increased risk of DVT. 

MTS is prevalent in women in their second to fourth decade of life [1]. 

Among the predisposing factors described are multiparity, hypovolemia, prolonged immobilization or travel, and pregnancy [1-2]. The use of contraceptives seemed to unmask underlying MTS in a case report series [3]. It is now believed that MTS is a progressive disease that could present in various clinical stages, from asymptomatic iliac vein compression (stage I) to the formation of a spur (stage 2), followed by a thrombotic phase or DVT in the LCIV [2]. 

The diagnosis of MTS is suspected in the presence of clinical manifestations suggestive of left-leg DVT. Swelling, pain, and leg heaviness are the classic symptoms. The most common physical examination findings are left leg erythema, edema, and local warmness. Consistent with these observations, our patient was a woman in her fourth decade of life who experienced typical symptoms of DVT after a prolonged journey. 

Massive venous edema could lead to an associated arterial insufficiency, known as phlegmasia cerulea dolens and gangrene, life-threatening complications of unrecognized extensive iliofemoral DVT. A thorough evaluation of symptoms and signs of compartment syndrome in MTS is crucial. Pulmonary embolism is a common concern in patients with MTS [4]. Respiratory complaints in the presence of hypoxia, persistent tachycardia, and hemodynamic compromise should prompt the consideration of thromboembolic pulmonary emergencies. 

Imaging studies of asymptomatic patients demonstrate the presence of left iliac vein compression in 25% of subjects [5]. Once symptoms occur, the diagnosis of MTS is best confirmed using radiographical modalities. Venous duplex ultrasound is employed as the initial most reliable noninvasive test to detect DVT in the iliac vessels, however, inter-operator reliability and obesity could limit the visualization of both the common and the external iliac veins [6] 

Computed tomography venography retains a similar sensitivity and specificity as Doppler ultrasound magnetic resonance venography would offer the advantage of identifying additional anatomic changes. Intravascular ultrasound (IVUS) has been proposed as one of the most important advancements in diagnosing and treating MTS. IVUS can illustrate stenosis, and it is an essential step prior to stenting [1].

The gold standard for the detection of MTS remains conventional venography that is commonly utilized when endovascular procedures are planned [7]. 

MTS treatment in different clinical stages remains challenging, but the management of MTS associated with DVT has evolved recently. Historically, acute VTE was treated with anticoagulation alone or surgical removal of the thrombus. The first CDT with stent placement in MTS was reported in 1995, and over time catheter-based procedures have replaced the traditional approach and are included in the current guidelines of Interventional Radiology [8-9]. The rationale for early intervention in iliofemoral DVT is that MTS patients are commonly young and remain with a high propensity to develop post-thrombotic syndrome. Post-thrombotic syndrome is associated with a decreased quality of life and an increased risk of disability secondary to the associated long-term complications: chronic edema, pain, and in extreme cases, venous ulcers [9]. CDT is superior and safer than the other options historically used. Compared with surgical thrombectomy, CDT does not necessitate laborious preparations in the operating room and provides faster recovery. Systemic thrombolysis enables thrombus lysis, but it is limited by the predefined dose of tissue plasminogen activator (TPA) administered, and by itself, systemic thrombolysis alone does not prevent recurrent DVT. 

The routine placement of an IVC filter prior to CDT is controversial, and current vascular surgery guidelines are not supportive of the pre-operative insertion of a retrievable filter [10]. The TPA is delivered locally at higher concentrations during CDT and can be administered for up to 48 hours with simultaneous heparin infusion. Ballooning angioplasty is recommended at 24 hours if there is residual persistent severe stenosis. After the successful removal of the thrombus is demonstrated via angiography, a stent is placed to facilitate the iliac vein outflow with the long-term goal to prevent recurrent DVT [9]. 

The most frequent concern with CDT is a higher risk of bleeding with a major bleeding rate of 8% [9]. A pre-procedure screening to assess for potential hemorrhagic complications remains paramount to identify patients who have contraindications for CDT thrombolysis and should be otherwise referred for thrombectomy. Major contraindications for CDT are active internal bleeding, cerebral infraction, neurological and eye procedures or head trauma within three months, and known intracranial tumor, aneurysm, or vascular malformation [11}.

Stent durability and flexibility are important predictors of in-stent re-thrombosis [11]. Still, the presence of a residual thrombus, an underlying hypercoagulable state, and the patient's compliance with anticoagulation may represent stronger prognosticators of in-stent re-thrombosis [12].

Anticoagulation treatment is an essential adjunctive therapy to maintain stent patency and prevent the reoccurrence of DVT. The patients are initially continued on unfractionated heparin following CDT, which is subsequently changed to oral anticoagulation for six months, and prescribed long-term aspirin to protect stent patency.

Our patient had a definitive treatment via thrombolysis followed by CDT with stent placement according to the guidelines; the patient experienced immediate symptom relief and early ambulation after the procedure.

## Conclusions

This case report highlights the importance of considering MTS in all patients with extensive DVT of the left lower extremity, specifically the LCIV. Life-threatening emergencies associated with MTS include phlegmasia cerulea dolens, arterial insufficiency, and gangrene. CDT in MTS prevents progression to post-thrombotic syndrome and associated long-term complications. Early CDT with stenting is currently considered an essential additional strategy to anticoagulation.
